# Pre-excited atrial fibrillation revealed at a very delayed age: case report

**DOI:** 10.1186/s12245-023-00506-z

**Published:** 2023-05-11

**Authors:** Thierno Hamidou Diallo, Raid Faraj, Safae Hilal, Myriam Lahraoui, Oualid Kisra, Fatima-Azzahra Benmessaoud, Nawal Doghmi, Ibtissam Fellat, Mohamed Cherti

**Affiliations:** 1 Clinical cardiology department, Cardiology Center, Mohammed V Military Instruction Hospital of Rabat, Mohammed V University, Rabat, Morocco; 2 Cardiology B department, Ibn Sina University Hospital of Rabat, Mohammed V University, Rabat, Morocco

**Keywords:** Wolff-Parkinson-White syndrome, Pre-excited atrial fibrillation, Electrocardiogram

## Abstract

**Background:**

Wolff-Parkinson-White (WPW) syndrome is a condition characterized by the persistence of an accessory pathway responsible for ventricular pre-excitation that can lead to symptomatic and potentially severe arrhythmias. Coexistence with atrial fibrillation is well known and not uncommon, exposing to potential degenerescence into ventricular fibrillation when atrial impulses are transmitted along the accessory pathway. WPW syndrome is most prevalent in younger patients and cases revealed after an advanced age have rarely been described in the literature.

**Case presentation:**

Here, we report a case of atrial pre-excitation first diagnosed at the age of 72 years that required external electrical cardioversion with a favorable outcome. The diagnosis was based on clinical and electrographic findings.

**Conclusions:**

WPW syndrome is a relatively rare cardiac disorder that can be a cause of sudden death, especially when combined with atrial fibrillation. Therefore, cardiologists have to consider this diagnosis in patients presenting clinical signs of arrhythmia with an electrical pattern of WPW.

## Introduction

Ninety-three years ago, Louis Wolff, Sir John Parkinson, and Paul Dudley White first described cases of 11 patients experiencing palpitations and a sinus rhythm with a combination of a short PR and a delta wave on the ECG [1]. This phenomenon was then named Wolff-Parkinson-White (WPW). Although patients with accessory pathways commonly present with either orthodromic or antidromic reciprocal tachycardia, atrial fibrillation (AF) is also present in one-third of patients with WPW syndrome [2]. During AF, several impulses reach the atrioventricular node, which normally blocks some of them through its decremental activity and thus controls the heart rate. In patients with an accessory pathway that leads in the anterograde direction, this protection is lost, and especially when the accessory pathway has a short refractory period, rapid conduction to the ventricle may result, making all the severity of AF in patients with pre-excitation [3].

Several publications have reported cases of pre-excited AF, particularly in young individuals, but few have reported such cases in advanced age.

Our paper was written according to the CARE guidelines [4].

## Case presentation

**Fig. 1:**
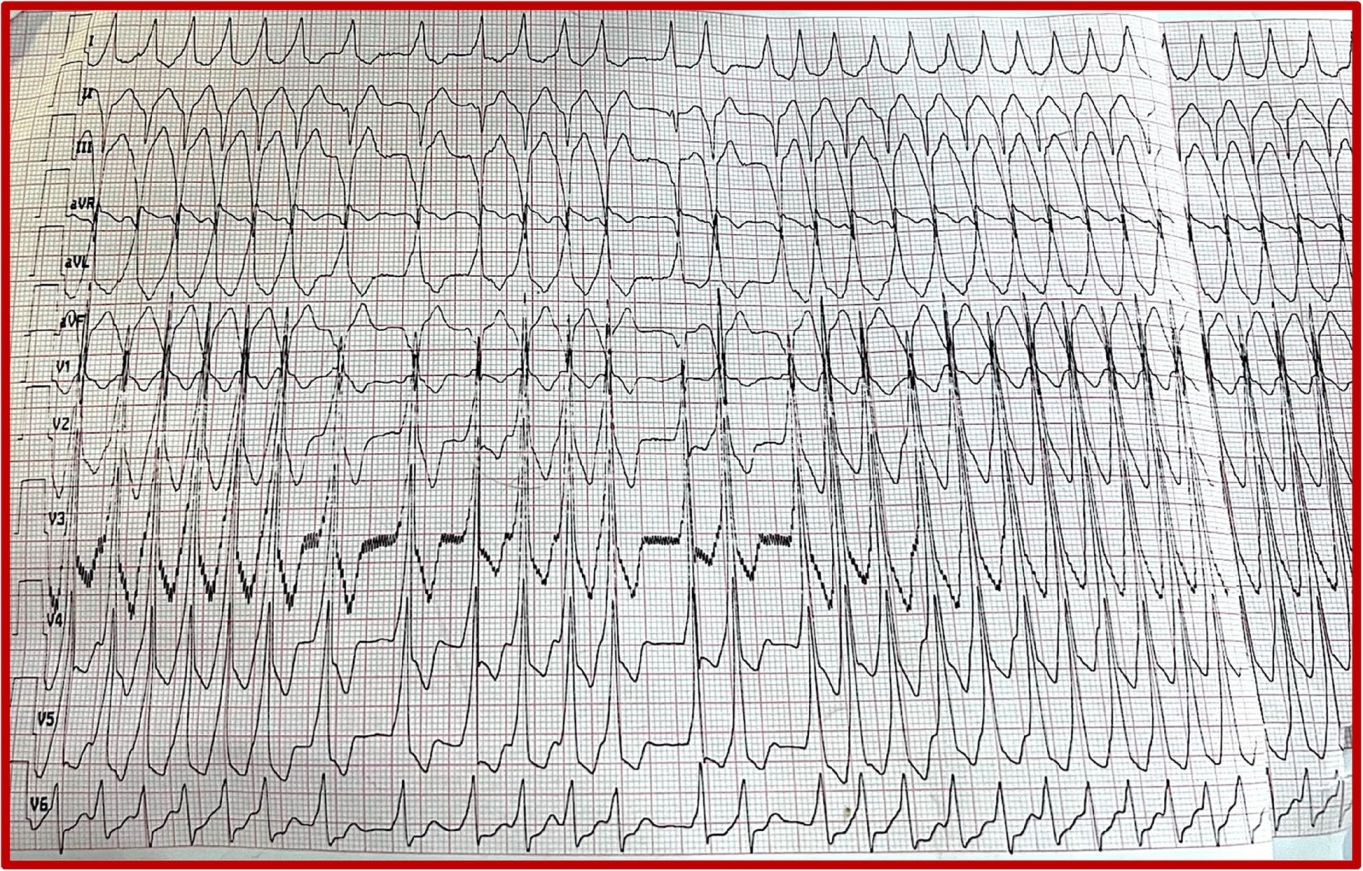
ECG findings: irregularly irregular wide-complex tachycardia changing its morphology from one complex to another without major changes in the electrical axis, creating an accordion-like appearance, suggestive of pre-excited AF

**Fig. 2:**
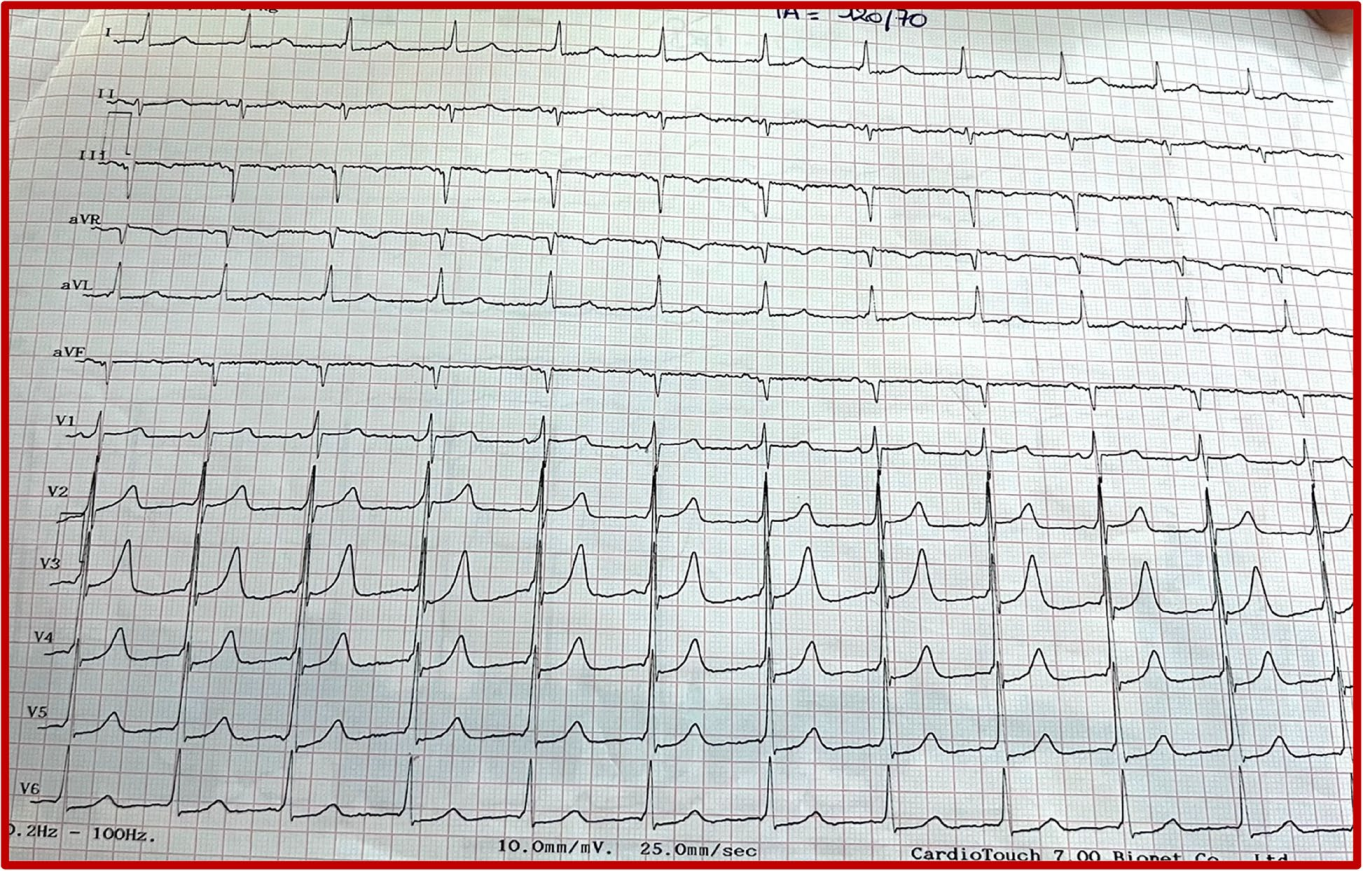
ECG findings after external electric shock: sinus rhythm with a short PR interval and a positive delta wave in V1, negative in inferior leads, and V1/DI ratio < 1, suggesting a left posteroseptal Kent bundle

**Fig. 3:**
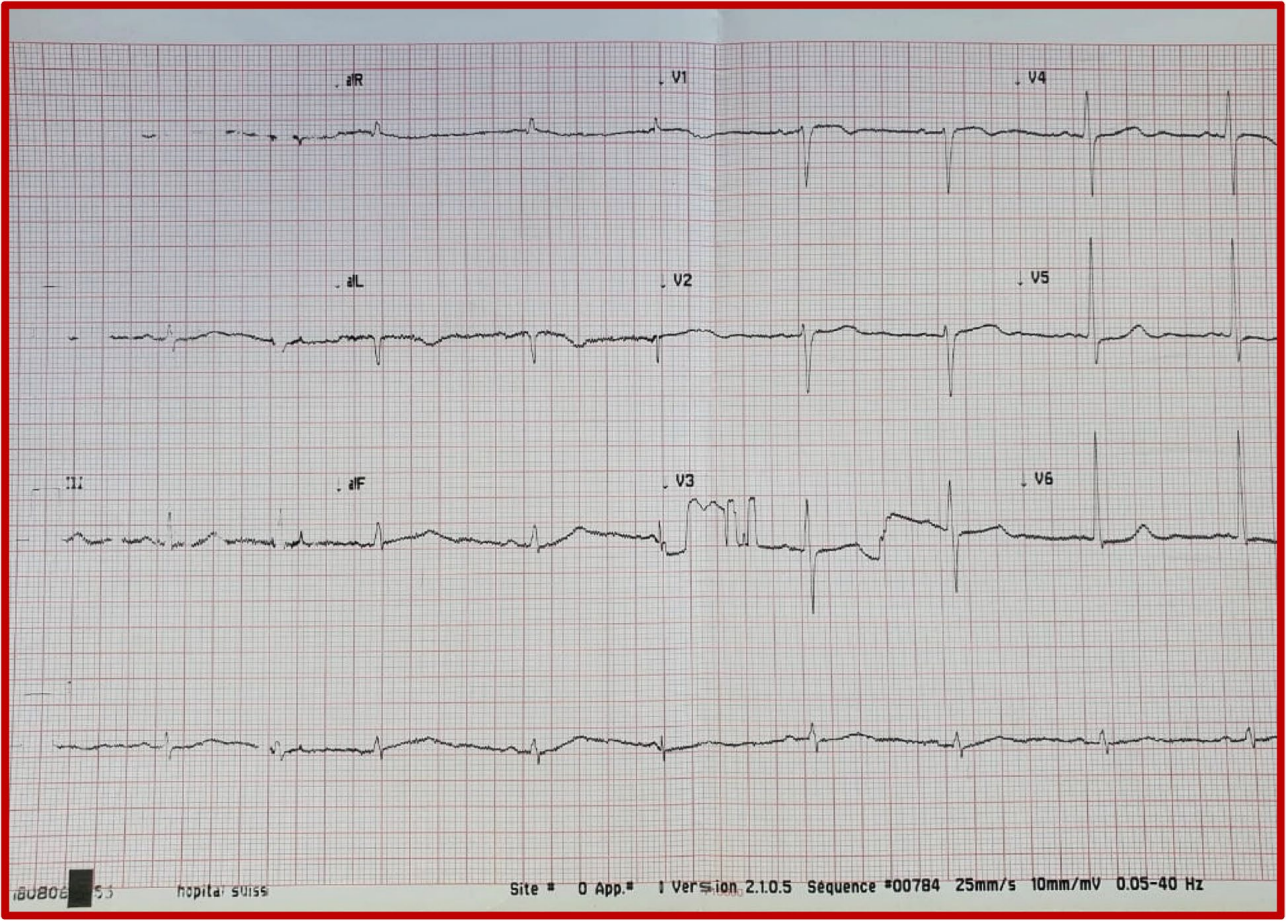
Previous ECG findings: no signs in favor of pre-excitation

**Fig. 4:**
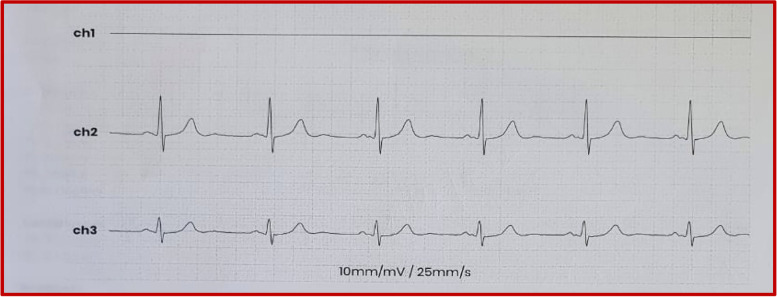
Previous Holter ECG findings: no signs in favor of pre-excitation

**Fig. 5:**
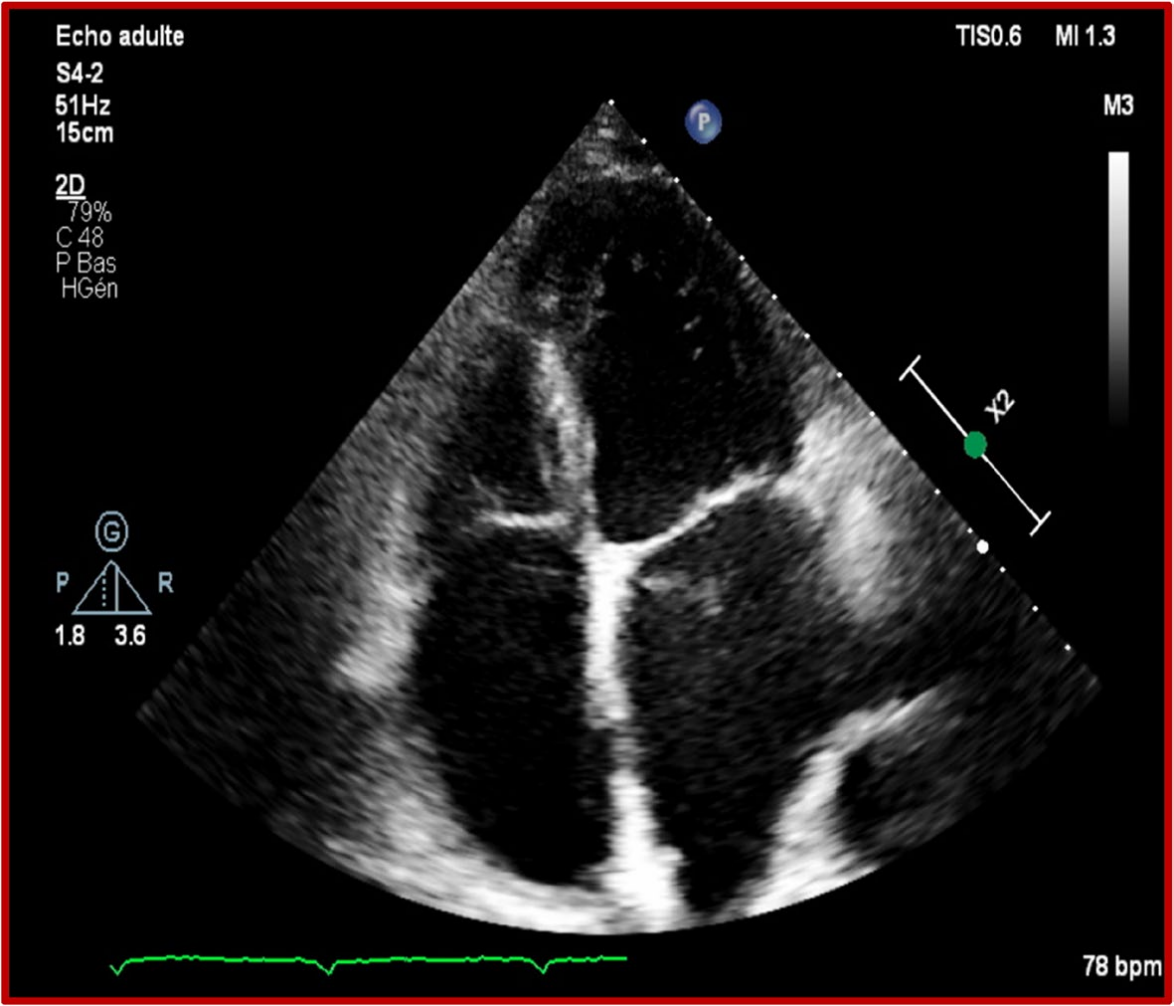
TTE findings: normal left ventricular function with a bi-atrial enlargement

We report the case of a 72-year-old patient admitted to the emergency department of Ibn Sina Hospital in Rabat for palpitations at rest of abrupt onset. He had a history of hemorrhagic rectocolitis under sulfasalazine. He was also followed for paucisymptomatic atrial hyperexcitability. His Glasgow Coma Scale was evaluated at 13/15. The patient presented hemodynamic instability with a blood pressure of 80/60 mmHg, a heart rate of 200 beats per minute, and cold extremities. The ECG revealed irregularly irregular wide-complex tachycardia changing its morphology from one complex to another without major changes in the electrical axis, creating an accordion-like appearance, suggestive of pre-excited AF (Fig. 1). The shortest pre-excited RR interval was 240 ms, denoting the potential for degeneration into ventricular fibrillation. Due to the unstable neurological and hemodynamic condition of the patient, an urgent external electric shock was administered at a power of 200 J which allowed the resolution of the tachycardia and the restoration of a sinus rhythm. The ECG in sinus rhythm showed a short PR interval with a positive delta wave in V1, negative in the inferior leads, and V1/DI ratio < 1 suggesting a left posteroseptal Kent bundle (Fig. 2). The clinical examination and the biological work-up were unremarkable. After analyzing his ECG (Fig. 3) and Holter ECG (Fig. 4) done 2 years ago, we did not find any sign in favor of pre-excitation. A transthoracic echocardiogram (TTE) found a normal left ventricular function, and a bi-atrial enlargement was noted (Fig. 5). The patient was discharged home 5 days after with flecainide and low-dose beta-blockers with a favorable evolution. An electrophysiological study with ablation of the accessory pathway was indicated, but the patient preferred to defer this to a later hospitalization.

## Discussion

Wolff-Parkinson-White syndrome is characterized by the presence of one or several atrioventricular accessory pathways, which links the atria and ventricles directly and bypasses the atrioventricular (AV) node, thereby predisposing patients to episodes of recurrent tachyarrhythmias associated with pre-excitation [5]. The prevalence of WPW should be discussed by distinguishing the WPW pattern from the WPW syndrome. Both are relatively uncommon, arising in less than 1% of the general population, with the WPW pattern being almost 100 times more frequent than the WPW syndrome [6]. Unlike our case, preexcitation is most often limited to young subjects without underlying heart disease. The discovery of preexcitation at an advanced age is unusual because most often patients present with symptoms such as palpitations, syncope, or episodes of anxiety during childhood or adolescence leading to the diagnosis of preexcitation either by surface ECG or electrophysiology study. Nevertheless, some patients remain asymptomatic throughout their lives even though they have overt preexcitation on ECG. On the other hand, preexcitation may be absent on the surface ECG, as it may be intermittent, latent, or even concealed [7]. Brembilla-Perrot et al. [7] reported in patients with arrhythmias, pre-excitation was found in 11% of patients with normal surface ECGs. The diagnosis of preexcitation on the surface ECG is considered in the presence of a short PR interval (< 0.2 s), a wide QRS (> 0.12 s in adults), and the presence of a delta wave [5]. The characteristics of this preexcitation depend on the location of the accessory pathway but also on the properties of the AV node and the his-Purkinje network [7]. Most non-latent accessory pathways are able to conduct anterogradely from the atrium to the ventricle and thus manifest as a WPW pattern. Nevertheless, some accessory pathways lead only retrogradely from the ventricle to the atrium; thus, the WPW ECG pattern is absent [6]. In our case, the patient was followed for supraventricular hyper-excitability for a long period of time, but his ECGs never revealed any signs of pre-excitation. Several hypotheses can be put forward to explain the absence of preexcitation on the surface ECG. The extent of ventricular preexcitation on the ECG in sinus rhythm depends on the quality of nodal conduction and the distance between the accessory pathway and the normal conduction system. The further the accessory pathway is from the sinus node and normal atrioventricular conduction, the less ventricular preexcitation is visible. The absence or intermittent disappearance of the delta wave may also be explained by precarious conduction in the accessory pathway, which as such would predict a good prognosis in the occurrence of arrhythmias including atrial fibrillation [2]. Patients with WPW syndrome have a high incidence of AF occurrence. Several hypotheses attempt to explain the mechanism of AF occurrence in patients with WPW syndrome. The disappearance of AF after the ablation of the accessory pathway suggests that a pathophysiological relationship exists between the presence of the accessory pathway and the development of AF [2]. The electrophysiological properties of the accessory pathway, including conduction in the anterograde or retrograde direction, play an important role in the genesis of AF. Indeed, there is palpable evidence that the coexistence of one or more accessory pathways and sustained orthodromic or antidromic tachycardia episodes plays an important role in the initiation of AF in patients with WPW syndrome. Architectural changes related to the presence of the accessory pathway, particularly at its atrial insertion, may cause micro reentry that may trigger AF. Moreover, the persistence of AF episodes even after ablation of the accessory pathway suggests that other mechanisms such as increased sympathetic activity and altered vagal tone, the influence of advanced age, and the intrinsic vulnerability of the atrial muscle, which itself may be aggravated by episodes of sustained tachycardia [8]. The management of patients with WPW syndrome associated with AF depends on the clinical presentation. In patients with hemodynamic instability, electrical cardioversion by an external electric shock is most commonly used. In hemodynamically stable patients, class IA, IC, or III antiarrhythmic drugs may be used. However, all drugs causing a slowing of AV conduction such as beta-blockers, adenosine, and digitalis should be avoided [9]. It is essential to emphasize that ablation of the accessory pathway remains the preferred therapy for the prevention of recurrent pre-excited AF, and medical therapy may be considered only in patients who are not suitable for, or refuse, ablation of the accessory pathway [10].

## Conclusion

Pre-excited AF is a rare situation that can be the sole and primary presentation of WPW syndrome. Cardiologists must be vigilant to this atypical presentation particularly when it occurs at a later age, in order to ensure appropriate management.

## Data Availability

Data sharing is not applicable to this article as no datasets were generated or analyzed during the current study.
